# Supplementary material for: Resveratrol and Resveratrol-Aspirin Hybrid Compounds as Potent Intestinal Anti-Inflammatory and Anti-Tumor Drugs
Source: Molecules. 2020 Aug 24;25(17):3849. doi: 10.3390/molecules25173849 (PMC7503224; doi:10.3390/molecules25173849)
Supplement: Supplementary file 1 [file molecules-25-03849-s001.pdf]

molecules

Supplementary Information

Resveratrol and Resveratrol-Aspirin Hybrid Compounds as Potent Intestinal Anti-Inflammatory and Anti-Tumor Drugs

Mohamed Salla [1], Vrajesh Pandya [1], Khushwant S. Bhullar [2], Evan Kerek [2], Yoke Fuan Wong [3], Robyn Losch [1], Joe Ou [1], Fahad S. Aldawsari [4,5], Carlos Velazquez-Martinez [4], Aducio Thiesen [6], Jason R. B. Dyck [2,3], Basil P. Hubbard [2] and Shairaz Baksh [1,3,7,8,9,*]

[1] Department of Biochemistry, Faculty of Medicine and Dentistry, Faculty of Pharmacy and Pharmaceutical Sciences University of Alberta, 113 Street 87 Avenue, Edmonton, Alberta T6G 2E1, Canada.; salla@ualberta.ca (M.S.); vrajeshk@ualberta.ca (V.P.); losch@ualberta.ca (R.L.); zo2@ualberta.ca (J.O.)

[2] Department of Pharmacology, 113 Street 87 Avenue, Edmonton, Alberta T6G 2E1, Canada.; bhullar@ualberta.ca (K.S.B.); kerek@ualberta.ca (E.K.); jason.dyck@ualberta.ca (J.R.B.D.); bphubbar@ualberta.ca (B.P.H.)

[3] Department of Pediatrics, Faculty of Pharmacy and Pharmaceutical Sciences University of Alberta, 113 Street 87 Avenue, Edmonton, Alberta T6G 2E1, Canada; yokefuan@ualberta.ca

[4] Faculty of Pharmacy and Pharmaceutical Sciences University of Alberta, 113 Street 87 Avenue, Edmonton, Alberta T6G 2E1, Canada.; aldawsar@ualberta.ca or fahad386@gmail.com (F.S.A.) velazque@ualberta.ca (C.V.-M.)

[5] Saudi Food and Drug Authority Laboratories, 3292 Northern Ring Road, Riyadh, 13312, Saudi Arabia.

[6] Department of Laboratory Medicine and Pathology, Faculty of Medicine and Dentistry, Faculty of Pharmacy and Pharmaceutical Sciences University of Alberta, 113 Street 87 Avenue, Edmonton, Alberta T6G 2E1, Canada; athiesen@ualberta.ca

[7] Departments of Oncology, Faculty of Medicine and Dentistry, Faculty of Pharmacy and Pharmaceutical Sciences University of Alberta, 113 Street 87 Avenue, Edmonton, Alberta, T6G 2E1, Canada

[8] Member, Cancer Research Institute of Northern Alberta and Women and Children's Health Research Institute

[9] BioImmuno Designs, Inc., 4560 TEC Centre, 10230 Jasper Avenue, Edmonton, Alberta T5J 4P6, Canada

* Correspondence: sbakshBID@gmail.com; Tel.: +01-780-239-0518

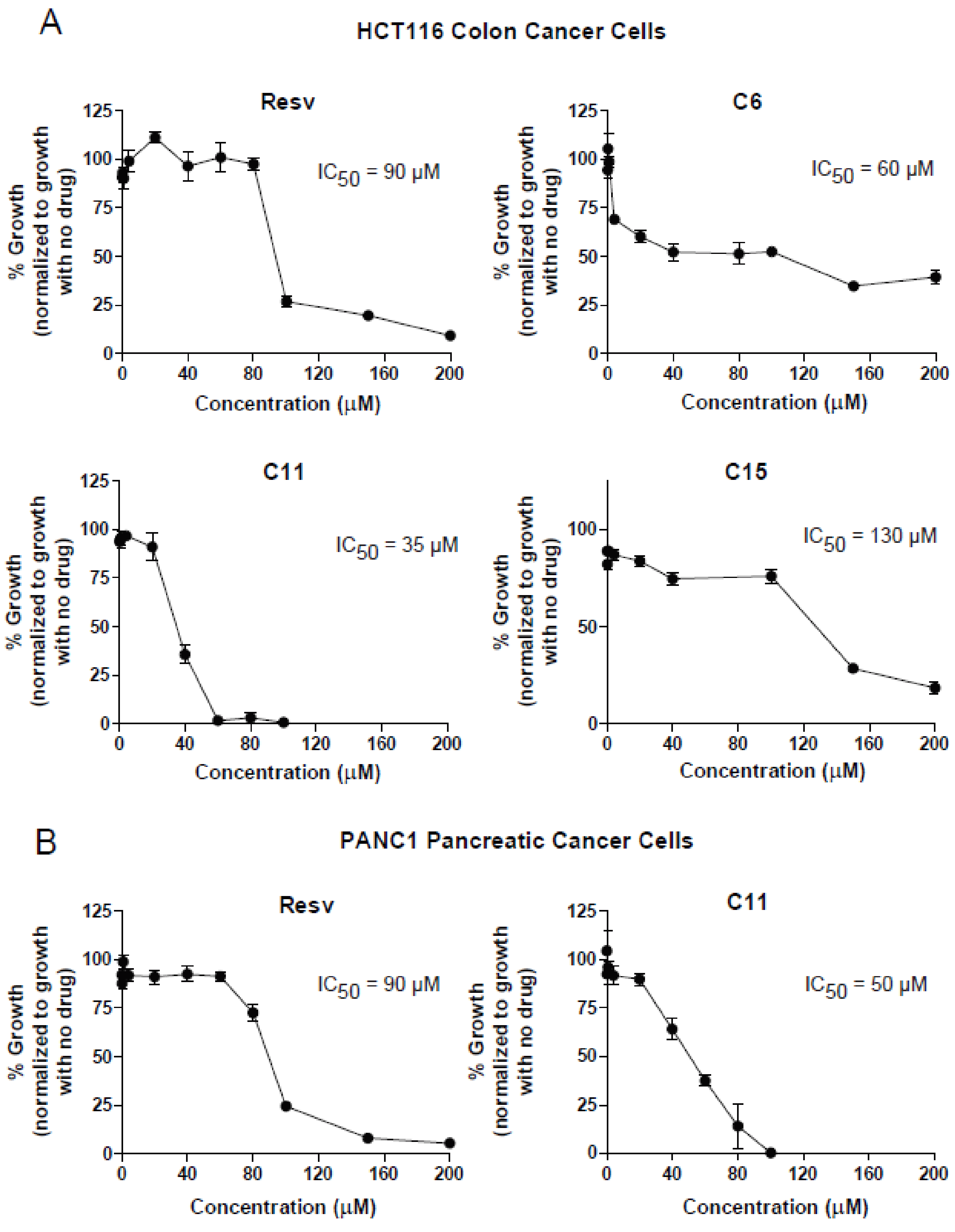

Supplementary Figure S1. IC_{50} curves for resveratrol and derivatives and their effect on cell viability (Related to Figure 1). Viability results using MTT assay with concentrations between 0 and 200 μM were used to generate the curves for effective compounds in (**A**) HCT-116 colon cancer cells and (**B**) PANC1 pancreatic cancer cells. Values were normalized against values with no drug addition and plotted. n = 4-5 replicates.

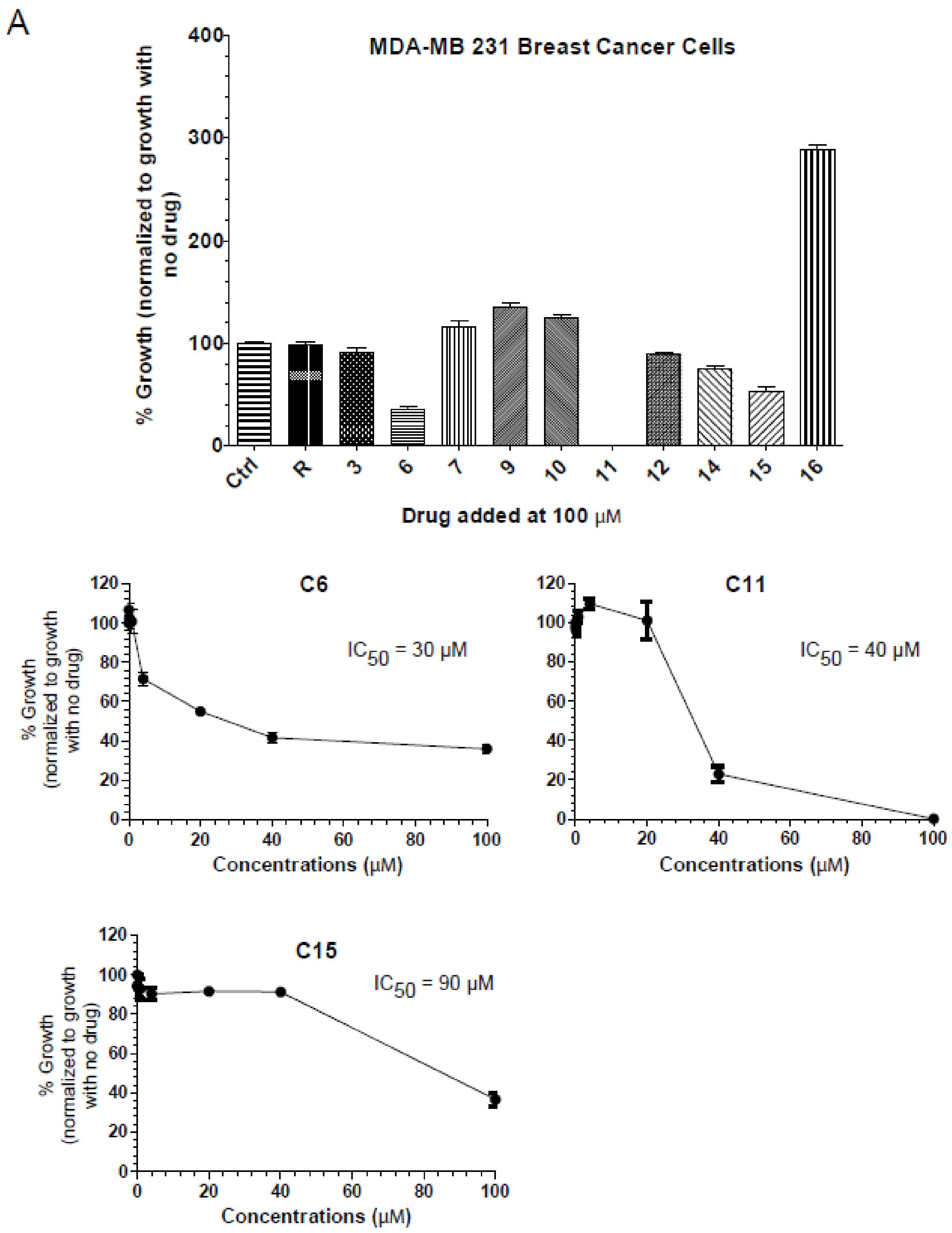

Supplementary Figure S2. Effect of resveratrol and derivatives on cell viability in 231 breast cancer cell line (Related to Figure 1). (**A**) Analysis of cell viability with resveratrol and derivatives at 100 μM in MDA-MB 231 breast cancer cell line with IC_{50} plotted for most effective compounds (concentrations between 0 and 100 μM). Values were normalized against values with no drug addition and plotted. $N = 8$.

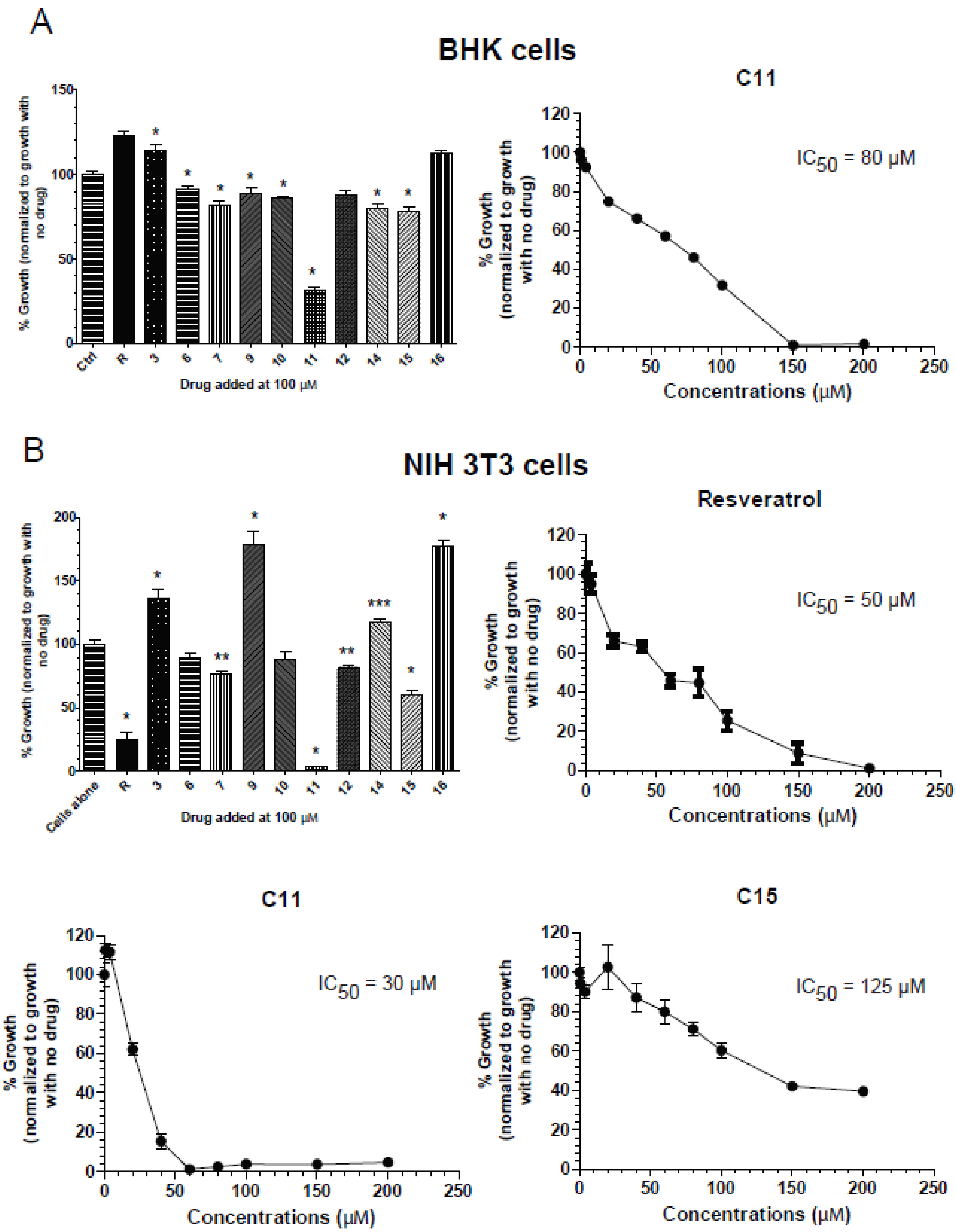

Supplementary Figure S3. Effect of resveratrol and derivatives on cell viability in (**A**) BHK (Baby Hamster Kidney) fibroblast cell line and (**B**) NIH 3T3 murine fibroblast cell line (Related to Figure 1). Analysis of cell viability with resveratrol and derivatives at 100 μM in both cell lines and IC_{50} plotted for most effective compounds (concentrations between 0 and 200 μM). Values were normalized against values with no drug addition and plotted. $N = 8$. * p-value < 0.0001; ** p-value < 0.001; "***" p-value < 0.01.

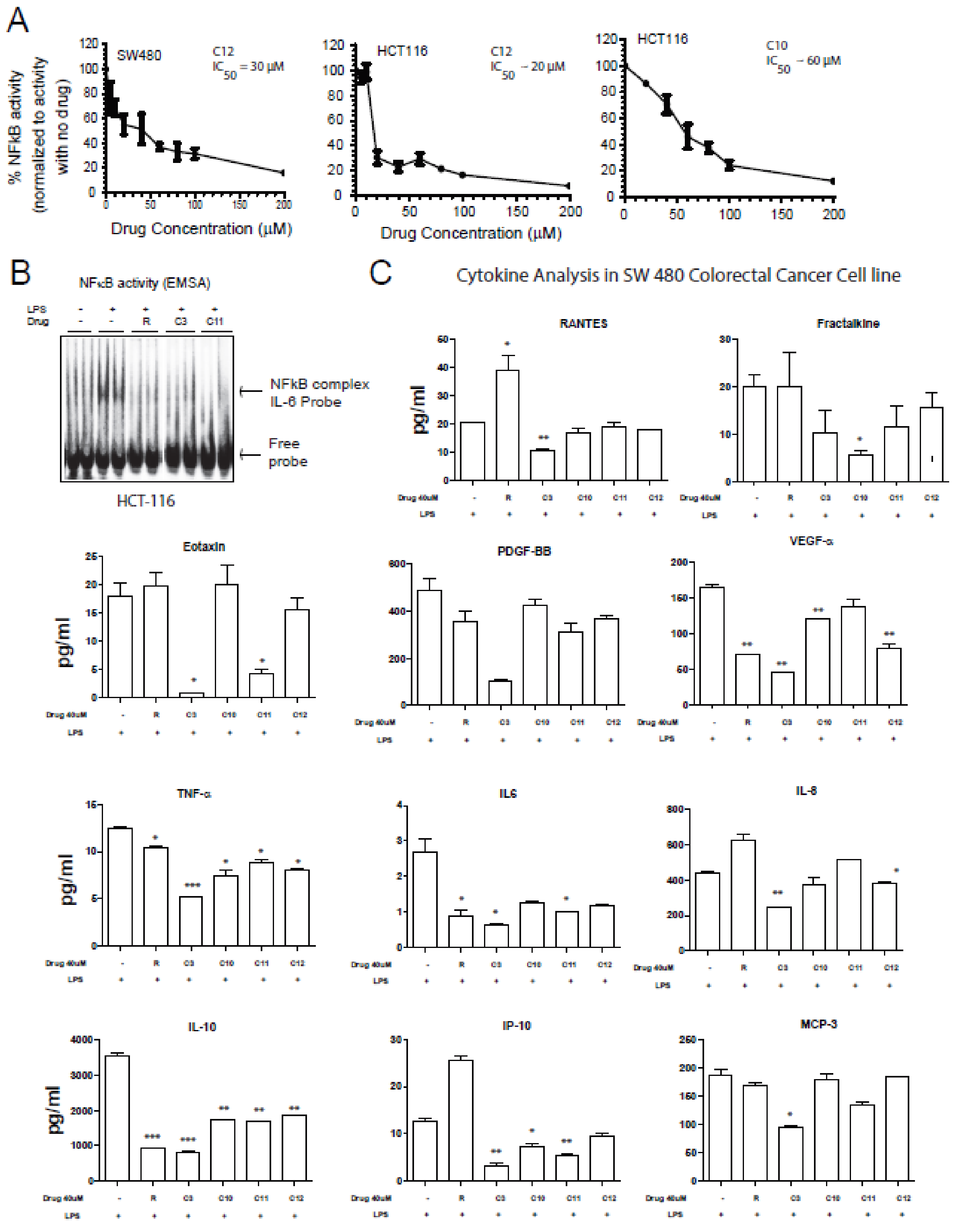

Supplementary Figure S4. Additional NFκB activity data in cultured cells. (A) NFκB activity was determined in SW 480 (left panel) and HCT116 (right panel) cells by a dual luciferase assay with firefly luciferase under the control of NFκB target sequence on IL-6 promoter as in Figure 2A (B) NFκB activity assessed by binding to the IL-6 promoter sequence DNA (AAATGTGGGATTTTCCCATGA) using the EMSA technique probe as in Figure 2D. *N* = 3 for each treatment and this is a second repeat of the one presented in Figure 2D. (C) Cytokine and chemokine levels were differentially reduced with resveratrol and derivatives. Briefly, SW 480 cells were allowed to grow in 6 well plates to 60% confluency, treated with drugs (100 μM) for 24 hrs and then stimulated with LPS (1.5 mg/ml) for 4 hrs. 500 μl of supernatant was collected and sent to Eve technologies. (https://www.evetechnologies.com/technology.php). *N* = 3-4. "*" *p*-value < 0.05, "**" *p*-value < 0.01, and "***" *p*-value < 0.001. If not stated, *p*-value > 0.05.

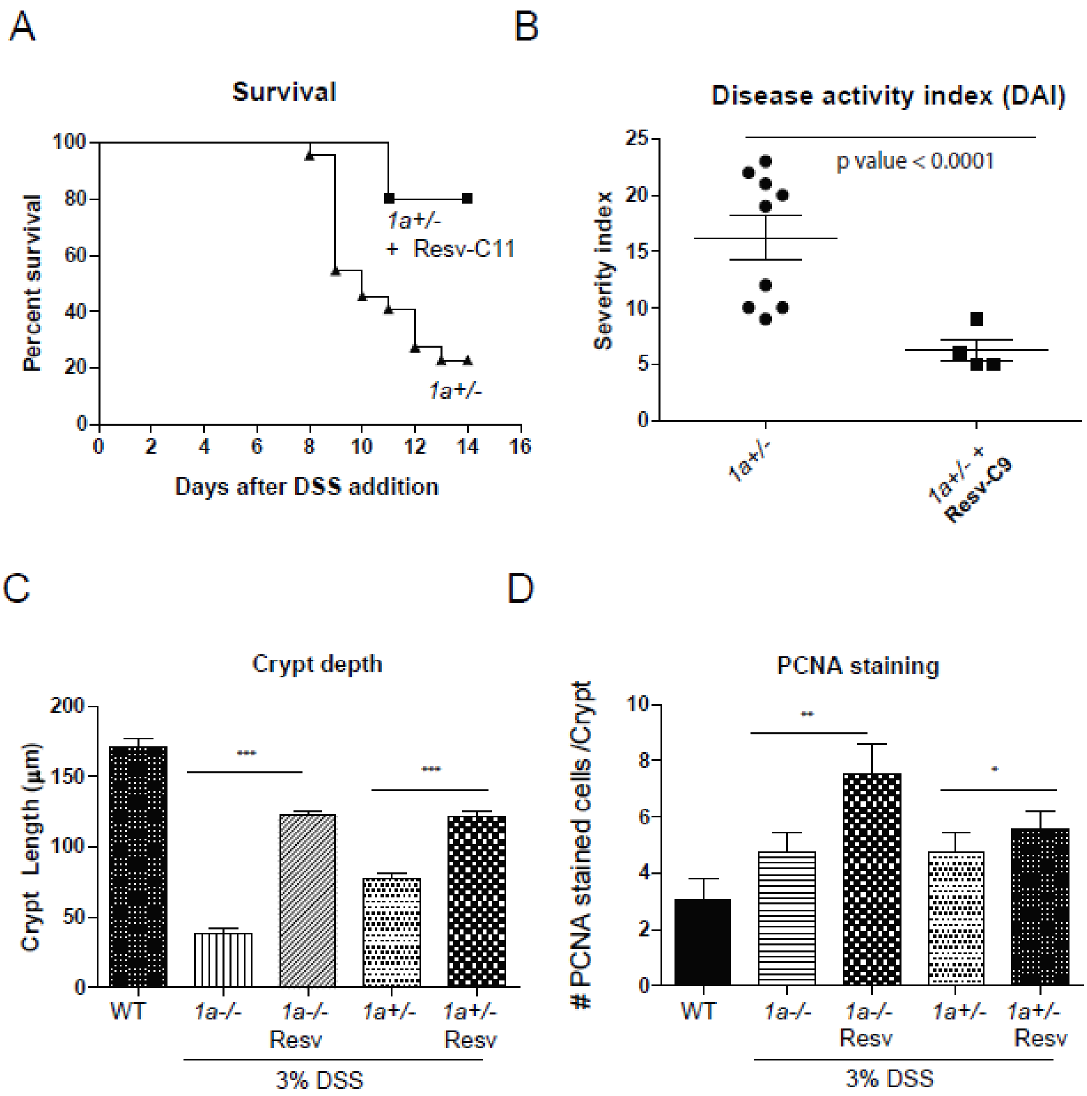

E

Parameter Measured / Genotype	Wild Type No Tx	Wild Type + DSS	Wild Type + DSS + Resv/Resv Derivatives	*Rassf1a*$^{-/-}$ No Tx	*Rassf1a*$^{-/-}$ + DSS	*Rassf1a*$^{-/-}$ + DSS + Resv/Resv Derivatives
Body Weight	100 %	88 ± 5%	93.6 ± 3%	100%	80 ± 6%	92 ± 4%
Food Consumption (per day)	5-6 g	2-3 g	3-5 g	5-6 g	1.5-2 g	3-4 g

Supplementary Figure S5: (**A**) C9 injected *Rassf1a*$^{+/-}$ knockout mice showed a higher survival rate and (**B**) less susceptibility to disease when given DSS in drinking water. Disease activity indices (DAI) accounted for several parameters including piloerection, bloated gut, movement, rectal bleeding, hunching, diarrhea, and weight loss. A numerical value of 1 to 5 was given with 5 being severe. If an animal was found dead 5 points added to previous day DAI. $N > 5$. (**C**) Crypt depth as determined by ImageJ analysis of crypts. Resveratrol fed animals showed both a more intact and deeper crypt in both knockout genotypes. (**D**) Measurement of PCNA positive proliferation in colonic sections. Resveratrol fed animals showed higher PCNA staining in both knockout genotypes indicative of enhanced tissue repair and wound healing in response to DSS induced injury. IHC staining was done using standard procedures. Percent PCNA staining was calculated using ImageJ software with software add on for DAP staining. N = 8-14; * p-value < 0.05, ** p-value < 0.01, and *** p-value < 0.001. If not stated, p-value > 0.05. (**E**) Table summarizing body weight and food consumption changes in non-treated, DSS treated and Resv/Resv derivative treated animals as indicated.

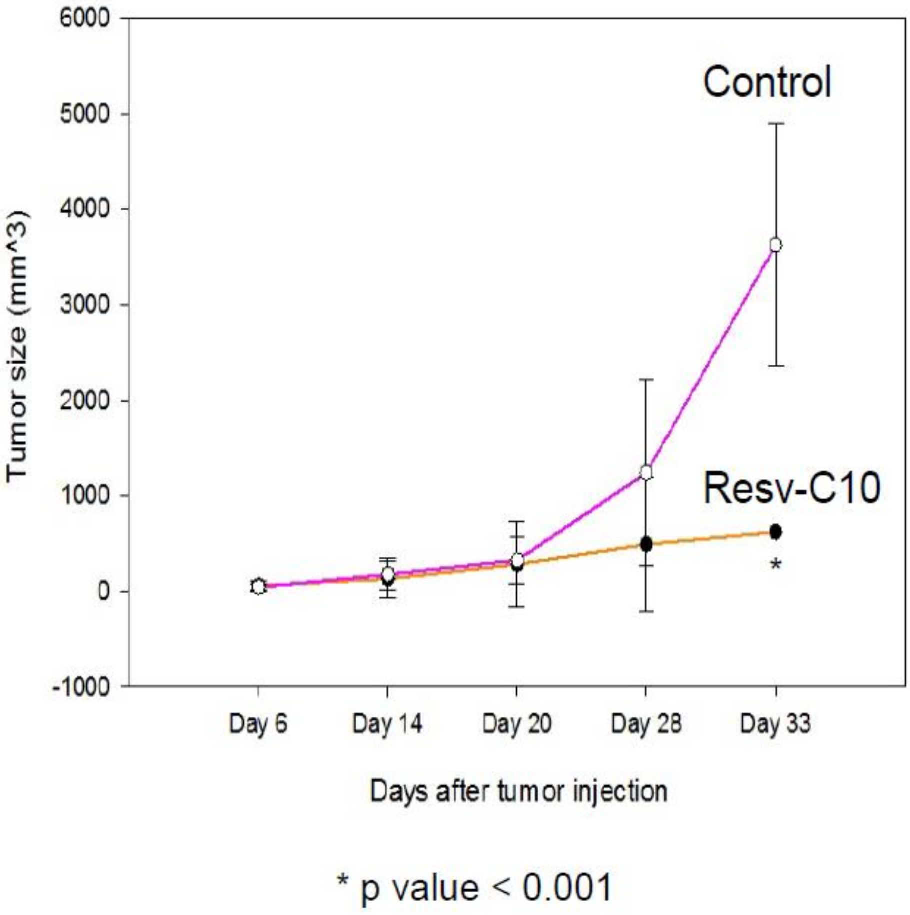

Supplementary Figure S6: Resveratrol-C10 can reduced tumor burden in a xenograft model. HCT116 colon cancer cells were injected subcutaneously into the flanks of athymic mice and tumor formation was monitored over 28 days. Resv-C10 was injected intraperitoneally at 0.3 mmol/kg (or 68.4 mg/kg) body weight every two days and examined for tumor formation as in Figure 6A. Shown is the time course of tumor formation with p value < 0.001 when compared to the control with no resveratrol-C10 ($n = 6$).
